# Case report: An adult intussusception caused by ascending colon cancer

**DOI:** 10.3389/fsurg.2022.984853

**Published:** 2022-09-09

**Authors:** Guowei Zhao, Wenjun Meng, Lian Bai, Qigang Li

**Affiliations:** ^1^ Department of Gastrointestinal Surgery, Yongchuan Hospital, Chongqing Medical University, Chongqing, China; ^2^ Department of Biotherapy, Cancer Center, West China Hospital, Sichuan University, Chengdu, China

**Keywords:** adult intussusception, colon cancer, computed tomography, colonoscopy, case report

## Abstract

Adults with bowel intussusception caused by malignant tumors are fairly uncommon. We presented a case of a 64-year-old woman whose intussusception was secondary to ascending colon cancer. A color Doppler ultrasonography of the abdomen revealed a low echo mass in the right middle abdomen. Physical examination and digital rectal examination were both unremarkable. Computed tomography (CT) revealed a concentric circle change in the colon, as well as the mesenterium and arteries. Electronic colonoscopy discovered the colonic giant proliferative lesions and stenosis. Adenocarcinoma with moderate differentiation was discovered after a biopsy. Then laparotomy showed intussusception and the tumor was located in the ascending colon. The postoperative pathological test revealed moderately differentiated adenocarcinoma in the right colon invaded the whole layer. After hospitalization, the patient was discharged without any complications. This case highlights that rational use of CT, endoscopy, and timely surgery combines an effective strategy for the treatment of adult intussusception.

## Introduction

Intussusception of the bowel is defined as the telescoping of a proximal segment of the gastrointestinal tract within the lumen of the adjacent segment. Adult intussusception represents 5% of all cases of intussusception and accounts for only 1%–5% of intestinal obstructions in adults (1, 2). Furthermore, adults with bowel intussusception caused by malignant tumors are fairly uncommon. To the best of our knowledge, there have only been a few reported cases of adult intussusception caused by colon cancer. Here, we presented a case of a 64-year-old woman whose intussusception was secondary to ascending colon cancer, which was diagnosed by abdominal computed tomography (CT) scan and colonoscopy.

## Case presentation

A 64-year-old woman was previously admitted to a local hospital 1 day ago for dizziness, hypodynamia, and loss of appetite. A color Doppler ultrasonography of the abdomen revealed a low echo mass in the right middle abdomen. For further diagnosis and treatment, she was treated in our hospital with a chief complaint of the color Doppler ultrasonography finding an abdominal mass.

**Figure 1:**
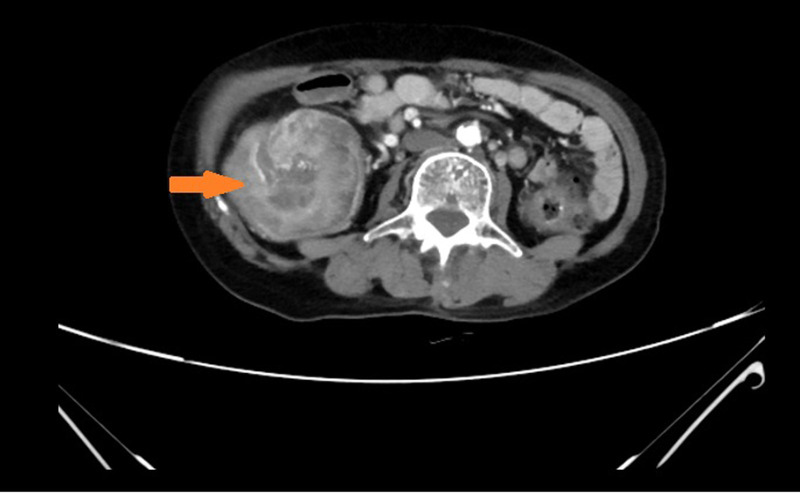
Computed tomography revealed ascending colonic intussusception.

**Figure 2:**
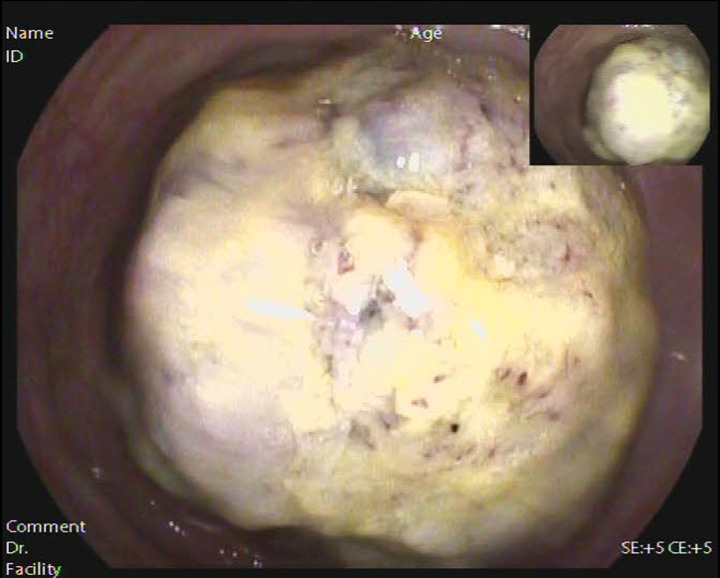
Electronic colonoscopy revealed a large proliferative lesion of the colon.

**Figure 3:**
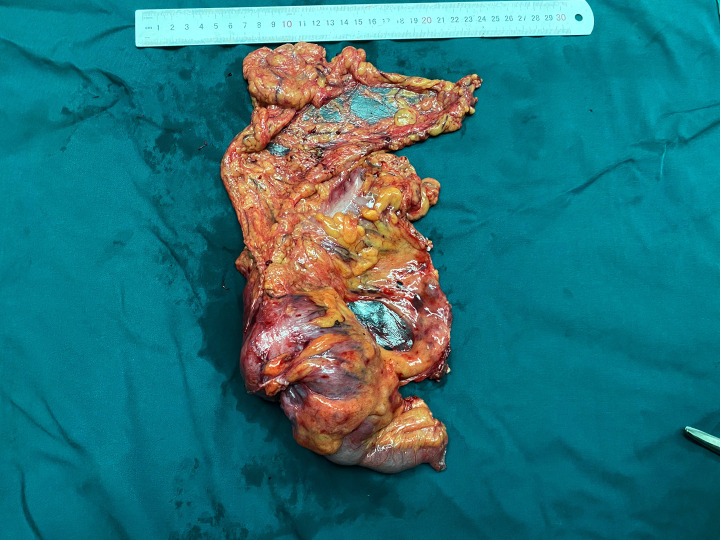
The surgical gross specimen.

Anemia signs in the upper abdomen were observed, and there were no other typical pathological signs. Although color Doppler ultrasound showed an abdominal mass, no obvious mass was touched during abdominal palpation. Hemoglobin concentration: 74 g/L (normal range: 110–150 g/L), albumin concentration: 27.2 g/L (normal range: 35–55 g/L), total protein concentration: 56.2 g/L (normal range: 60–80 g/L). Based on the above information, our first clinical considerations for the abdominal mass were as follows: intestinal space-occupying lesions (possibly malignant tumors). Enhanced CT revealed a concentric circle change in the colon, as well as the mesenterium and arteries (Figure 1). The segment of the colon wall was edematous and thickened. To conclude, it was considered a tumor in the colon. Following an electronic colonoscopy, it was discovered that the colonic giant proliferative lesions had stenosis (Figure 2). Adenocarcinoma with moderate differentiation was discovered after a biopsy. After receiving the biopsy results, our medical group discussed and concluded that the patient should be treated with an operation, so elective laparoscopic right hemicolectomy was performed. During the operation, radiological examination results were confirmed. Finally, we found the tumor was located in the ascending colon, where the intestinal canal was partially inserted into the distal ascending colon. The range of lymph tissue we scavenged during the operation included the root of the ileocolic artery, the surrounding of the middle colon artery and vein, the root of the superior mesenteric artery, and the lower edge of the pancreas. We cut the transverse colon and ileum at 15 cm away from the tumor, and then anastomosed the ileum and transverse colon side-to-side. The tumor was about 7.0 cm × 6.0 cm × 2.0 cm, mass-type, hard, invaded the serosa, and occupied 2/3 circles of the intestine (Figure 3).

**Figure 4:**
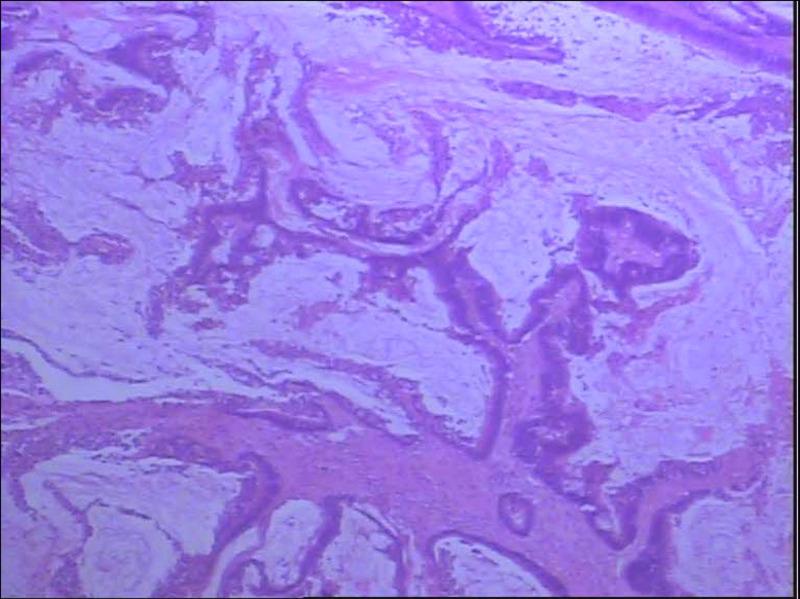
Postoperative pathological result revealed moderately differentiated adenocarcinoma in the right colon with full-thickness invasion (magnification power: 40×).

Gross examination of the resected specimen showed that the intestinal canal was 23 cm long, which contained the ascending colon tumor with a volume of 7.5 cm × 6.0 cm × 3.5 cm. The cross-section was gray-white and invaded membrana serosa. The pathological result showed a moderately differentiated adenocarcinoma invaded the whole layer; lymph nodes near the colon were negative (Figure 4). Immunohistochemistry: Ki67 (about 60%, +), MLH1 (+), MSH2 (+), MSH6 (+), PMS2 (+), Her-2 (weak +). The patient recovered smoothly and was discharged 12 days after the operation. She was followed up for 1 year, and there was no recurrence.

## Discussion

The incidence of adult intussusception is rare. While it is common in children, adult intussusception accounts for only 1% of intestinal obstruction and less than 5% of all cases of intussusception (1, 2). Different from children with apparent symptoms such as abdominal pain, abdominal mass, and bloody stool, adults have various symptoms, which may be acute, intermittent, or chronic. Most affected adults develop pre-diagnosis episodes of intermittent abdominal pain and vomiting (3). In this case, the symptoms and signs had no obvious specificity, so it was difficult to diagnose and may have caused a delay in treatment.

In adult intussusception, 60% of cases are caused by malignant and benign neoplasms; the remaining non-idiopathic cases are usually caused by postoperative adherences, Crohn's disease, infections, intestinal ulcers, or Meckel diverticulum (2, 4). To help guide treatment decisions, it is important to diagnose the organic intussusception lesion. Enema or colonoscopy can detect and reduce intussusception, which is conducive to the qualitative diagnosis of organic lesions (5). Patients diagnosed with colonic or ileocolic intussusception are usually accompanied by tumors, and there are usually no clear clinical signs of acute abdomen. In these settings, preoperative endoscopy can be performed to confirm the presence of pathology and/or cancer (6). Compared with ultrasonography, barium enema, and colonoscopy, CT is the most accurate preoperative diagnostic method. In a recent report by Hong et al. (7), abdominal CT accurately diagnosed intussusception in 77.8% of patients. Eventually, this patient was diagnosed with intussusception by CT and confirmed benign and malignant lesions by colonoscopy biopsy. Therefore, when the clinical manifestations are difficult to diagnose, CT should be routinely performed to make a definite diagnosis. In addition, colonoscopy can also be used to distinguish benign or malignant lesions for ileocecal and colonic intussusception (8).

At present, surgery is still the main treatment for adult intussusception. It is determined according to the length of the affected intestine in patients with intussusception. In other words, if the affected portion of the small intestine is not extensive, surgery cannot reduce the intussusception. If resection of a long segment of the bowel is required, intraoperative reduction can be attempted to reduce the resection length (9). Because malignant diseases are highly correlated with intussusception in adults, surgical resection without reduction should be limited to primary malignant disease. However, when the small bowel is the only tract involved, the reduction of bowel intussusception can be attempted because of its lower rate of association with malignancy (10). Primary adenocarcinoma is the main cause of colon intussusception. Considering the high incidence of primary adenocarcinoma, it should be resected without reduction in colonic intussusception (7). When tissue diagnosis is unavailable, intussusception is located in the colon and is highly likely to be malignant lesions, it is advisable to follow the oncology principle of colon resection as a precautionary measure to provide the best opportunity for curative resection. Most surgeons believe that laparotomy is necessary for the treatment of adult intussusception because of the high incidence of underlying malignancy colon intussusception, and the inability to differentiate benign or malignant in enteric intussusceptions (6). In a recent review article, it is reported that when there are signs and symptoms of acute abdomen, abdominal exploration is the gold standard; when there are signs of septic shock and peritonitis, emergency exploration is mandatory (6). Some surgeons believe that laparoscopic surgery can also be selected. The choice of laparoscopic surgery rather than open surgery depends on the clinical situation of patients and the experience of surgeons (11). Laparoscopic surgery cannot be selected in the following cases: (I) severe heart, lung, liver, or renal insufficiency; (II) severe coagulation dysfunction that is difficult to correct; (III) severe intestinal adhesion; and (IV) diffuse peritonitis with intestinal obstruction. In this case, a biopsy through enteroscopy confirmed that it was moderately differentiated adenocarcinoma. Therefore, laparoscopic right hemicolectomy was chosen, and total resection without reduction is performed to avoid potential intraluminal seeding or venous tumor dissemination. Eventually, right colon cancer was diagnosed, so the monitoring and evaluation of this case were also based on the right colon cancer.

In conclusion, although the optimal treatment for adult intussusception remains controversial, the definitive treatment depends on the underlying etiology and location. For stable patients without emergency indications for surgery, a thorough preoperative diagnostic evaluation and medical optimization should be performed. Because adult intussusception is highly correlated with malignant tumors, unless small bowel is the only tract involved or is proved to be benign by tissue diagnosis, it should follow the oncology principle of colon resection as a precautionary measure. When clinical manifestations are difficult to diagnose intussusception, CT is the most accurate method for a definite diagnosis. In addition, colonoscopy is recommended only when intussusception is located in the ileocecal and colon, which can not only assist the diagnosis, but also provide a reference for surgical methods.

## Data Availability

The original contributions presented in the study are included in the article/Supplementary Material, further inquiries can be directed to the corresponding author/s.
